# Editorial: Alleviating age-related disease burden

**DOI:** 10.3389/fragi.2026.1828980

**Published:** 2026-04-28

**Authors:** Matthew Halma, Sidra Hassaan, Jack A. Tuszynski

**Affiliations:** 1 Open Source Medicine Foundation, Tallinn, Estonia; 2 Department of Physics, University of Alberta, Edmonton, AB, Canada; 3 DIMEAS, Politecnico di Torino, Turin, Italy

**Keywords:** age related diseases, aging population, disease burden, healthy ageing, Preventive medicine

The life expectancy of humans has increased tremendously--up to an average of 30 years in 1870, up to 73.6 years in 2022, and is projected to be 78.1 years by 2050 (Scott, 2023; Vollset et al., 2024)--and, most of it, due to long-run changes in the quality of living and health conditions (Keshavarz et al., 2023). These gains are deeply associated with population-wide increases in safe water, sanitation, hygiene, nutrition, and wider gains in living standards, which have decreased the mortality rates of infectious diseases and extended life expectancy across the life course (Scott, 2023; Wolf et al., 2023). Current global projections suggest that life expectancy will continue to increase in most regions of the world through 2050, but that the rate of change will be lower than that experienced over the decades leading up to the COVID-19 pandemic (Vollset et al., 2024).

Age-related disease (ARD) is an important topic for our times, given the historically unprecedented lifespans humans are living, which are expected to increase even further (Scott, 2023; Vollset et al., 2024). ARDs are a significant contributor to global morbidity and mortality, particularly in developed nations where the population is increasingly aged (Gianfredi et al., 2025; Kallestrup-Lamb et al., 2024). As individuals grow older, the risk of developing a variety of chronic conditions, such as cardiovascular diseases, neurodegenerative disorders, and metabolic syndromes, increases dramatically (Gianfredi et al., 2025; Kallestrup-Lamb et al., 2024). These diseases not only diminish the quality of life but also place a substantial burden on healthcare systems and economies (Gianfredi et al., 2025; Kallestrup-Lamb et al., 2024; Kocot et al., 2024). The concentration of healthcare expenditure in the latter decades of life creates of individuals that is not just a medical challenge, but a societal and economic one (Kallestrup-Lamb et al., 2024; Kocot et al., 2024). Countries with pension programs will have to account for longer life expectancies and, hence a greater burden on the social support system (Groenew et al., 2025). Optimizing lifestyle factors for chronic disease prevention pays greater dividends the earlier these changes are implemented, so it is important to shift healthcare towards preventative medicine and education regarding lifestyle modifications aimed at longer health spans, not just lifespans (Gianfredi et al., 2025; Cocchi et al., 2025). Expenditures on chronic diseases, especially over several decades, present economic challenges for the financing of healthcare (Kallestrup-Lamb et al., 2024; Kocot et al., 2024).

One facet of ARDs is that these have emerged in developed countries, after major investments in sanitation and public health, which coupled with increased overall affluence have eliminated or greatly reduced early causes of death such as infant mortality and infectious diseases (Wolf et al., 2023). In order to maintain the solvency of national healthcare systems beset by ARDs, healthcare costs must come down, which is where lifestyle changes can enter into a national strategy (Gianfredi et al., 2025; Kocot et al., 2024; Cocchi et al., 2025). The work of our contributing authors to this research topic includes projections for age related disease burden for pulmonary arterial hypertension (PAH), gout, as well as general age-related diseases with examples regarding both national and global developments.

However, the biological aging process is driven by complex molecular and cellular mechanisms, including genomic and metabolic instability, telomere attrition, epigenetic alterations, and mitochondrial dysfunction (López-Otín et al., 2023). Recent advances in aging research have begun to unravel these processes, offering new insights into potential interventions that could delay or prevent the onset of age-related diseases (Suda et al., 2024; Tartiere et al., 2024). Interventions that target the underlying mechanisms of aging, rather than just the symptoms, hold promise for extending healthy lifespan and reducing the overall burden of ARDs (Suda et al., 2024; Tartiere et al., 2024).

Even though age-related disease and biological aging are closely related, they are different. Biological aging is the gradual time-associated decrease in physiological resistance and homeostatic ability, which is caused by genomic instability, epigenetic changes, mitochondrial dysfunction, and cellular senescence (López-Otín et al., 2023; Tartiere et al., 2024). Age-related diseases, in turn, are clinical conditions the incidence and severity of which increase with age, but are not equal to aging, as they are also influenced by environmental, behavioral, socioeconomic, and genetic factors (Keshavarz et al., 2023; Kopp, 2024). In this regard, aging must be perceived as a significant biological context and risk intensifier to chronic disease, but not as a disease. The difference is noteworthy to the current research topic since its key findings focus on the burden, distribution, and consequences of diseases in older populations and not on the direct measurement of the aging process (Gianfredi et al., 2025; Kopp, 2024).

Through prioritization of age-related diseases, the public health systems will have the opportunity to allocate resources to prevention, early risk detection, and maintain functional health in old age (Gianfredi et al., 2025). Biomarkers of aging and digital surveillance are currently under consideration to detect increased biological deterioration prior to the manifestation of a detectable disease, and eventually augment risk stratification and prophylactic care (Yamada, 2025; Lu et al., 2025). Nevertheless, the clinical translation of these tools is not fully developed, and the problem of validation, standardization, and interpretability continues to restrict their application to population health and clinical care (Herzog et al., 2025; Furrer and Handschin, 2025). This is why, in the current editorial, biomarkers, wearables, analytics, and coaching must be considered as a set of prospective implications into a larger construct of healthy-aging, as opposed to the immediate interventions under testing by the articles included in this research topic (Menassa et al., 2025; Cen et al., 2025). These tools are discussed as future-oriented implications rather than direct findings of the topic.

For ARDs, it may make sense to see health coaches and personal trainers as being a more proactive response, whereas physicians and nurse practitioners can be relied on in case of disease development (and emergency room doctors and nurses in the case of acute health challenges). To adopt a proactive stance towards aging, this requires establishing rigorously the relationship between lifestyle factors and ARDs (Gianfredi et al., 2025; Cocchi et al., 2025). Some insurance companies have started to incentivize proactive health behaviours, such as checkups, regular fitness, or healthy eating (Salmani et al., 2025; Petracca et al., 2025). This approach acknowledges that there is a relationship between lifestyle factors and health (Salmani et al., 2025; Petracca et al., 2025).

Fitness and diet coaching has long struggled with a lack of acknowledgement from the medical system. While their approach may not treat disease in the same way that a course of antibiotics treats and infection or a surgery removes the threat of a ruptured appendix, their clients, if they are to be consistent, will have a significant impact on their health trajectory over the years (Gianfredi et al., 2025; Cocchi et al., 2025).

It is time for medicine to include evidence based lifestyle changes to deal with the challenges of aging populations (Gianfredi et al., 2025; Cocchi et al., 2025). This approach will also help to ease acute shortages of doctors by reducing disease incidence and severity when the disease is impacted by lifestyle (Gianfredi et al., 2025; Cocchi et al., 2025). An aging population can represent a crisis if we are unprepared, but foresight can help to create a healthy future for older adults (Gianfredi et al., 2025).

The aim of the current Research Topic was to cover promising, recent, and novel research trends in the field of aging and age-related disease prevention. Areas to be covered in this Research Topics included.

- Epidemiological and economic analyses of age-related disease impact
- Reviews and meta-analyses on aging biomarkers and interventions
- Studies on the biological and molecular basis of interventions targeting aging
- Development and application of therapeutics that slow biological aging
- Predictions of the impact of aging interventions on population health and economics.

The studies in this editorial address the issue of aging not by directly measuring biological aging, but rather by examining the diseases, burden of disability, and care demands that are particularly salient within older populations and aging societies. That is, they are relevant to aging because of the age-stratified increase in incidence, multimorbidity, functional decline, dependency, and economic burden associated with longer-lived populations (Vollset et al., 2024; Gianfredi et al., 2025; Kopp, 2024). This difference is significant: the research topic mostly reports clinical and societal burden in terms of aging populations, whereas the rest of the geroscience literature can be used to understand why these burdens increase with age and why prevention-based strategies are still appealing (López-Otín et al., 2023; Tartiere et al., 2024; Kopp, 2024).

The papers that are included in this editorial are particularly focused on conditions and care burdens that are prominent in later life. Xu et al. focused on analyzing pulmonary arterial hypertension in adults aged 60 years and above, which puts the analysis directly in the context of an aging population (Xu et al.). Yang and Liu assessed the burden of gout among patients aged 70 years and above, once again focusing on the disease patterns in older age (Yang and Liu, 2025). Yang et al. evaluated the burden of ageing-spectrum diseases in China and found a significant rise in the number of disability-adjusted life years per age group, aged 65 years and above, thus attributing ageing of the population to the escalating burden of chronic diseases (Yang et al.). Le Toullec et al. focused on the burden and needs of family caregivers to the elderly, as a critical downstream impact of aging societies: with a growing number of individuals living with chronic illnesses and dependency, the issue of caregiver burden also constitutes a meaningful part of the overall public health problem (Le Toullec et al.). Combined, these studies have been related to aging based on older-age case distributions, age-stratified burden, and the impact of longer survival with chronic disease on society as opposed to direct measures of aging biology itself.

In brief, diseases of aging are expected to rise in incidence, motivating healthy aging strategies to lower the incidences of age related diseases (Vollset et al., 2024; Gianfredi et al., 2025). For gout, incidence correlates with socio-demographic index (SDI) (Yang and Liu, 2025), and is significantly higher in males than in females. PAH shows a complex pattern of incidence, with rates rising between 1990 and 2000, then decreasing up to 2017, and increasing thereafter. Despite the changes in incidence, mortality from PAH remained steady from 1990 to 2006, increasing to 2011, and is currently decreasing, despite increased incidence (Xu et al.).

A broad examination of age-related diseases in China identified a 34% increase in DALYs among individuals aged 65 years and above (Yang et al.). To counter the rise, the authors identify several items which can help to lower the burden of disease in older individuals: reducing particulate matter pollution, smoking, and high sodium diets (Yang et al.).

Beyond the burden on individuals and health systems, there is also considerable burden on caregivers, whether professional caregivers such as nurses, or family members (Le Toullec et al.). The authors performed a scoping review on the burdens and needs of family caregivers for the elderly, finding heterogeneity in the reliability and validity of the scales used in the literature to measure caregiver support; finding only two scales valid (Matsuda, 1999; Thornton and Travis, 2003) out of 29 identified.

Current scales to measure caregiver burden are inadequate to measure caregiver’s engagement and resources, which can help to minimize burnout and improve care for elderly people.

Although the overall discourse lends relevance to biomarkers, proactive measures, and healthy-aging models, the papers included in the editorial tend to focus on the disease burden, age-specific epidemiology, and caregiver load, and do not directly test interventions aimed at modifying biological aging. Based on this, such prevention-related elements are to be interpreted as implications and future perspectives, instead of the results that have been determined by the topic itself. Even though Blue Zones have been dominant in popular and academic debates on longevity, their interpretation is controversial since some researchers have criticized the accuracy of demographics, age verification, and how exceptional-longevity regions have been identified, whereas others have defended the rigor of the validation procedures in at least some of these populations (Austad and Pes, 2025; Caruso et al., 2025). That is why the idea of Blue Zones can be better viewed as a controversial source of assumptions about environmental and lifestyle factors affecting healthy aging instead of solid evidence of causal lines to extraordinary longevity.

In conclusion, healthy-aging strategies might be adopted to decrease disease burden in the long term, especially in diseases that have long preclinical stages, like dementia, in which early risk modification can affect subsequent outcomes (Gianfredi et al., 2025; Sperling et al., 2011). Simultaneously, cases that are used to justify the existence of exceptional longevity, like Blue Zones, should be viewed with caution. One of the relatively simple ways of affecting the health outcomes is to change the diet toward a plant-based diet with the primacy of whole foods, which are traditional to the local region of the population concerned (Pes et al., 2022). In Okinawa, one of the identified blue zones, 0.8% of people born during the 1900 birth cohort reached the age of 100, compared to 0.3% in the USA and 0.1% in the UK (Poulain et al., 2004). Moreover, the long-lived populations in the Blue Zones point to a way of healthy aging whereby their elderly population retains a high degree of autonomy and independence in their everyday lives, many still keeping up leisurely activities such as reading and sports (Fastame et al., 2018). These populations continue to provide informative data when it comes to formulating hypotheses about diet, environment, social connectedness, and lifelong behavior, but their demographic interpretation has also been contested due to the issue of age validation and methodological consistency. Therefore, instead of considering Blue Zones as conclusive evidence, it is better to consider them as an extension of a larger and ever-changing body of literature on the determinants of healthy aging.

Overall, the current topic is best argued as an analysis of the burden of disease and care among aging populations instead of a direct analysis of interventions that alter biological aging. It has contributed by demonstrating how populations that live longer are experiencing an increasing burden of chronic disease, disability, and caregiving, and why these facts bolster the argument in favor of prevention-based, healthy-aging policy. Continued work into the future must be in linking epidemiologic burden research with validated biomarkers, mechanistic geroscience, and pragmatic interventions, but the current body is much more focused on providing evidence about the magnitude and impact of the challenge itself.
